# Prospective 3D Fat Navigator (FatNav) motion correction for 7T Terra MRI

**DOI:** 10.1002/nbm.5283

**Published:** 2024-10-26

**Authors:** Krzysztof Klodowski, Ayan Sengupta, Iulius Dragonu, Christopher T. Rodgers

**Affiliations:** ^1^ Wolfson Brain Imaging Centre University of Cambridge Cambridge UK; ^2^ Department of Psychology, Royal Holloway University of London London UK; ^3^ Siemens Healthcare Ltd Camberley UK

**Keywords:** 7T, FatNav, motion correction, navigator, prospective, UHF

## Abstract

Ultra‐high field (7T) MRI allows scans at sub‐millimetre resolution with exquisite signal‐to‐noise ratio (SNR). As 7T MRI becomes more widely used clinically, the challenge of patient motion must be overcome. Retrospective motion correction is used successfully for some protocols, but for acquisitions such as slice‐by‐slice scans only *prospective* motion correction can deliver the full potential of 7T MRI. We report the first implementation of prospective 3D Fat Navigator (“FatNav”) motion correction for the Siemens 7T Terra MRI. We implemented a modular Sequence Building Block for FatNav and embedded it into the vendor's gradient‐recalled echo (GRE) sequence. We modified the reconstruction pipeline to reconstruct FatNav images online, coregistering them and sending motion updates to the host sequence online. We tested five registration algorithms for performance and accuracy on synthetic FatNav data. We implemented the best three of these in our sequence and tested them online. We acquired T_1_ and T_2_* weighted brain images of healthy volunteers correcting every other image for motion to visualise the effectiveness of online motion correction. Data were acquired with and without head immobilisation. We also tested performance while correcting every measurement for motion. Our implementation uses a 1.23 s 3D FatNav acquisition module and delivers motion updates in less than 3 s, which is sufficient for motion updates every few *k*‐space lines in typical scans. Corrected images are crisper with fewer visible motion artefacts. This improved sharpness is reflected quantitatively by an increase in the variance of the image Laplacian which is 1.59 x better for corrected vs uncorrected images. Profiles across the cerebral falx are 33% steeper for corrected vs uncorrected images. Prospective FatNav improves GRE image quality in the brain. Our modular Sequence Building Block provides a simple method to integrate motion correction in 7T MRI pulse sequences.

## Graphical abstract

We implemented prospective 3D FatNav motion correction for Siemens 7T Terra MRI scanners. We compared registration algorithms in synthetic data. We tested in healthy volunteers: corrected images were visually crisper, and had improved sharpness measured by increased variance of the image Laplacian and by sharper profiles across the cerebral falx.

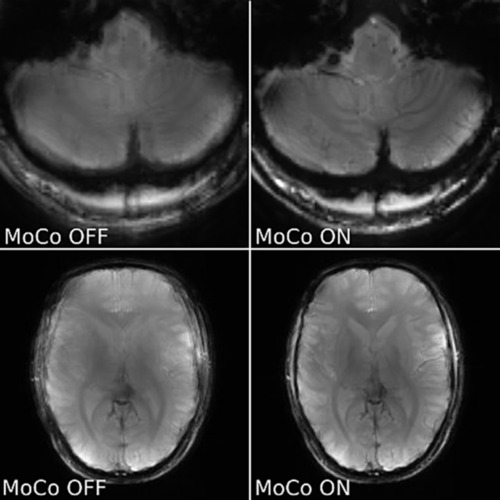

**Abbreviations**
CCC: Concordance Correlation Coefficient
FatNav: Fat Navigator
ICE: Image Calculation Environment
MARS: Measurement and Reconstruction System
MoCo: Motion Correction
RMSE: Root Mean Square Error
SSIM: Structural Similarity Index
vNav: volumetric navigator

## 1 INTRODUCTION

Ultra‐high field (7T) MRI offers exquisite signal‐to‐noise and sub‐millimetre spatial resolution. Clinical applications of 7T MRI are becoming more common. Yet, motion artefacts remain a serious challenge, especially at 7T [1, 2, 3]. A recent analysis of 42,874 motion logs from UK Biobank 3T fMRI scans revealed that 4% of scans were rejected because of head motion [4]. This cohort was generally healthy adults between 40 and 69 years old. For patients, the situation is more pressing. It is estimated that up to 20% of MRI scans have to be at least partially repeated [5].

Many motion correction tools have been created, often requiring additional hardware [3, 6, 7, 8, 9]. Image‐based motion correction works well, but so far on the 7T Terra scanner it is limited to retrospective motion correction [10]. Although retrospective motion correction can substantially improve image quality, prospective motion correction can be superior, e.g. it allows correction of through‐slice motion in slice‐by‐slice 2D imaging which is not possible retrospectively. Even for 3D imaging, prospective correction performs better for undersampled scans [11]. We note that prospective motion correction based on volumetric navigators has been reported for 3T [12] and 7T Philips scanners [13].

We aimed to implement a prospective motion correction method for brain scans on the Siemens 7T Terra. Our ultimate intended target is for clinical research scans in patients with dementia, who are likely to make intermittent movements during scanning and who in our experience struggle to comply with marker‐based tracking systems. In particular, 7T T_2_*‐weighted imaging or quantitative susceptibility mapping (QSM) shows strong benefits for detecting small cerebral microbleeds which are biomarkers of neurodegeneration [14]. In Parkinson's disease type II mixed resting and action tremor is observed in half of the patients, and in half of them the tremor is reemergent with a typical time lag of 5 s–20 s between the events [15]. For our intended applications, we require motion updates every 5 s to allow correction of motion or reacquisition of some *k*‐space lines or image volumes without unduly increasing overall scan time. The technique should be modular and flexible enough for easy implementation in various sequences.

To achieve this goal, we have created a new framework for *prospective* 3D Fat Navigator (FatNav) [16, 17] motion correction. We implemented this as a modular Sequence Building Block (a modular component that can be added to pulse sequences on Siemens MRI scanners) and added it to the vendor's gradient‐recalled echo (GRE) sequence. We also modified the vendor's Image Calculation Environment (ICE) online reconstruction chain to reconstruct navigators online, coregister them to a reference image and send motion updates to the sequence in real‐time. We describe the implementation, an assessment of the performance and accuracy of coregistration codes, performance in synthetic data derived from human volunteer scans and in vivo head scans.

## 2 MATERIALS AND METHODS

### 2.1 GRE‐FatNav sequence

**FIGURE 1:**
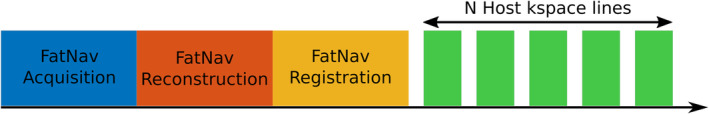
Schematic of product GRE sequence with FatNav navigator embedded every N host k‐space lines.

We created a Sequence Building Block implementing a 3D fat navigator according to the protocol of Gallichan et al [10]. We added this module to the vendor's GRE sequence, opting to embed fat navigators every N × TR, where N is the number of *k*‐space lines acquired for each measurement (whole scan) (Figure 1), i.e. N = 256 or 192 (for T_1_ and T_2_* host images, respectively). Such a choice gives motion updates once every 43 s for T_1_‐weighted images and once per 167 s for T_2_*‐weighted imaging. Detailed host sequence parameters are specified in Table 1. For the initial tests, the sequence was programmed to correct for motion in odd‐numbered measurements but not in even‐numbered measurements which were always acquired in the original frame of reference. This allowed us to compare corrected vs non‐corrected images directly for the human scans. In these tests, we acquired a whole image between each FatNav but updated the sequence after every other navigator. Further tests were carried out with more frequent motion updates. The acquisition of each 3D FatNav volume took 1.23 s as described in the retrospective implementation by Gallichan et al [10]. Other navigator parameters were: 128 × 128 × 88 matrix, 256 × 256 × 176 mm^3^ FOV, 3.2 ms TR, 1.49 ms TE, 6/8 partial Fourier, 4 × 4 GRAPPA acceleration and 7° nominal FA. A binomial 1–2‐1 RF pulse with inter‐lobe spacing of 0.5 ms was used for the fat‐selective excitation. The images presented here were acquired with motion updates once per measurement. This added an extra 8.61 s (i.e. 1.23 s every 43 s) for T_1_‐weighted imaging and 6.15 s (i.e. 1.23 s every 167 s) for T_2_*‐weighted imaging scans.

**TABLE 1:** Sequence parameters for the main (host) sequence.

	T_1_ GRE (2D)	T_2_* GRE (2D)
FOV	300x300 mm	300x300 mm
Matrix	256x256	192x192
TR	168 ms	872 ms
TE	10 ms	20 ms
FA	25°	51°
Slice thickness	5 mm	3 mm
Number of slices	12	10
Number of measurements	7	5
Acquisition time	5 min	14 min

### 2.2 Image reconstruction

**FIGURE 2:**
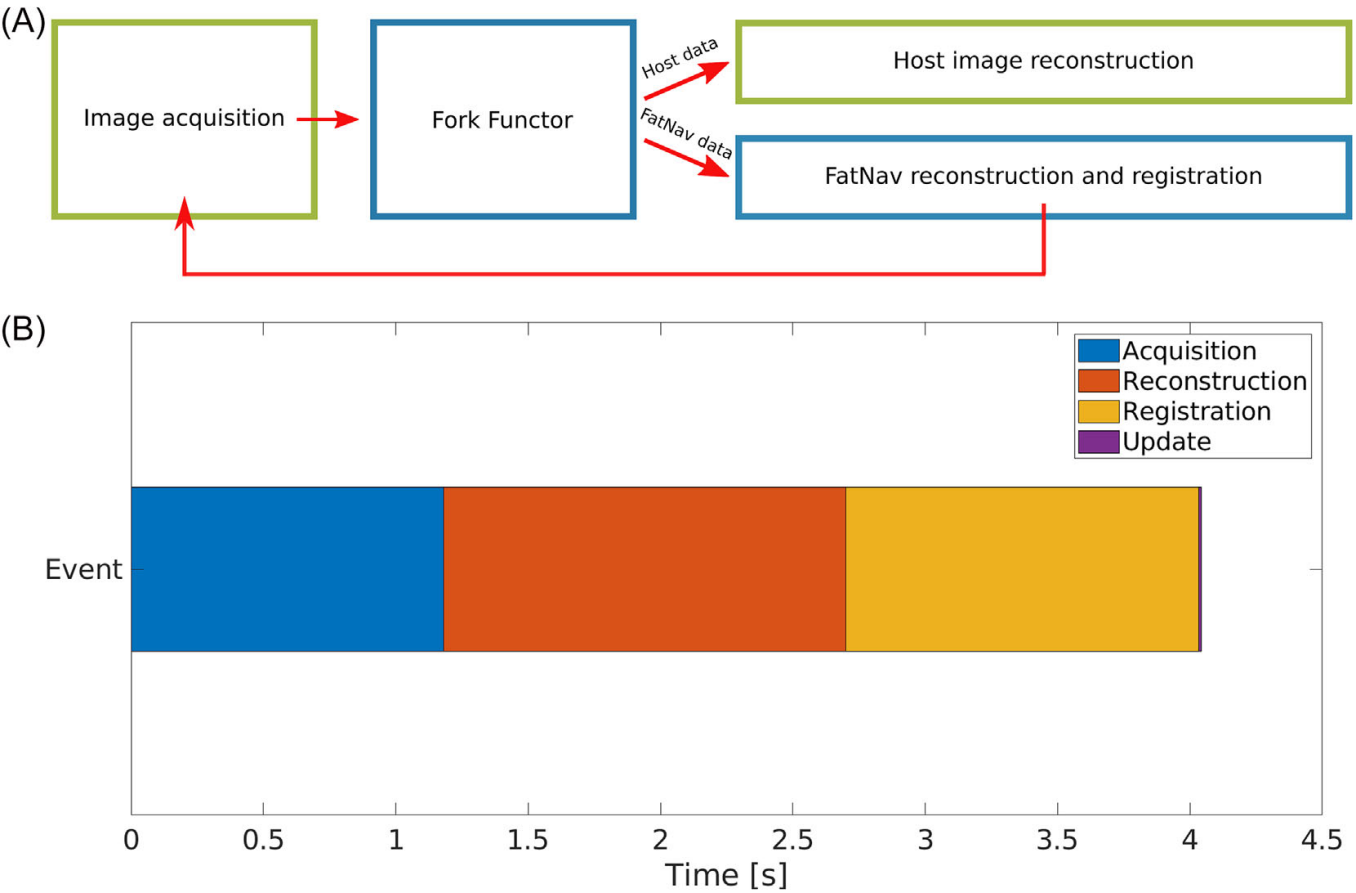
(A) Schematic of the modified Siemens ICE reconstruction pipeline. (B) Timing diagram of particular events of motion correction method. This figure depicts average times for the simpleITK registration algorithm.

Data were reconstructed using a modified version of the vendor's online ICE reconstruction pipeline (Figure 2A). The navigator data were tagged by the sequence with an “RTFEEDBACK” flag; the Fork Functor (a module in the reconstruction chain) separated them from the host sequence data so as not to interfere with the unaltered host sequence reconstruction pipeline. The 3D navigator volumes were reconstructed in ICE and then sent to a registration functor to compute motion parameters. The computed parameters were sent to the sequence and used to adjust the frame of reference for the acquisition of the next host sequence measurement in our test protocols [18]. The updated rotation matrix and translation offsets internally update imaging gradients and therefore positioning of the next host image acquisition.

### 2.3 Online registration of 3D FatNav volumes

**FIGURE 3:**
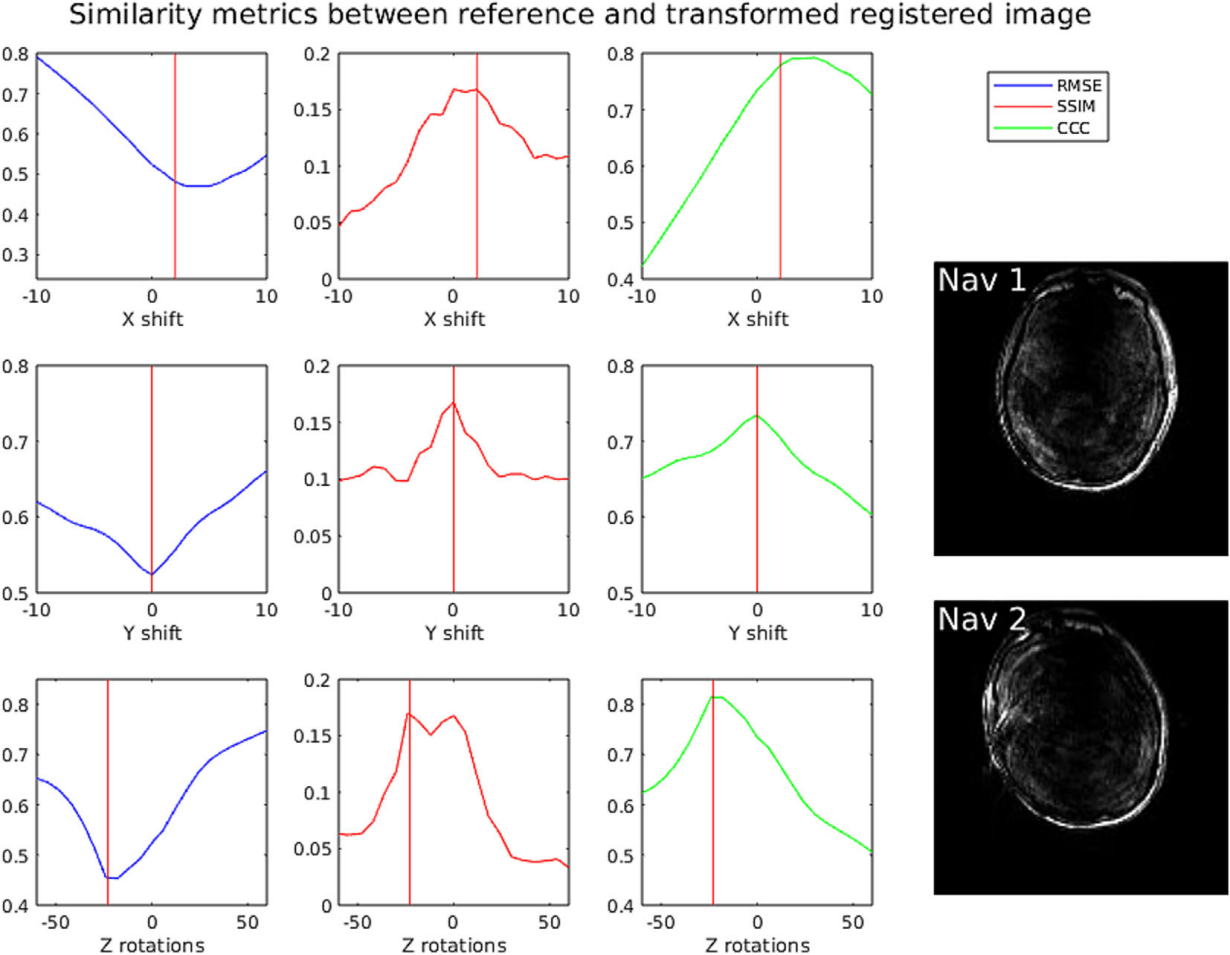
Comparison of behaviour of various similarity metrics under translation and rotations of a rotated and shifted 2D fat navigator image (“Nav 2”) with respect to a fixed reference scan (“Nav 1”). Red vertical lines indicate the ground truth values based on manual registration. Abbreviations: RMSE – root mean square error metric, SSIM – structural similarity metric, CCC – concordance correlation coefficient metric.

Rapid registration of 3D navigator volumes is a crucial part of navigator‐based *prospective* motion correction. 3D FatNav images, shown for example in Figure 3, have relatively low spatial resolution, often include pronounced artefacts and have different typical features compared to a normal (water) image. These factors make registration of FatNavs challenging [19]. We therefore assessed possible registration algorithms: imregtform (included in the Image Processing Toolbox, Mathworks Inc, Nattick, MA, USA), simpleITK (using rigid Euler3DTransform, mean squares similarity metric, gradient descent optimiser and linear interpolator) [20], Greedy [21], the vNav registration algorithm [22] and our own custom registration algorithm implemented in C++ directly in ICE. The latter is a 3D registration algorithm intended for rapid rigid registration of 3D FatNav volumes. The algorithm selects smaller sub‐volumes and searches for a centroid coordinates and rotation angles using concordance correlation coefficient (CCC) as a similarity metric [23], which for a pair of images x and y is defined as:

(1) $$\rho_{C}=\frac{2s_{\mathrm{xy}}}{s_{x}^{2}+s_{y}^{2}+{\left(\bar{x}-\bar{y}\right)}^{2}},$$

in which $s_{\mathrm{xy}}$ is the covariance between pixels in images x and y, $s_{x}^{2}$ and $s_{y}^{2}$ are the variances of pixels in images x and y, respectively and $\bar{x}$ and $\bar{y}$ are the mean values of the pixels in images x and y, respectively.

We also considered using the structural similarity index (SSIM) [24], defined as:

(2) $$S\left(x,y\right)=f\left(l\left(x,y\right),c\left(x,y\right),s\left(x,y\right)\right),$$

where $l\left(x,y\right),\;c\left(x,y\right),\;s\left(x,y\right)$ are comparison functions for luminosity, contrast and structure.

The third metric we took into account was root mean squared error (RMSE):

(3) $$\text{RMSE}=\sqrt{\frac{1}{n}\sum_{i=1}^{n}{\left(x_{i}-y_{i}\right)}^{2}},$$

where n is the number of pixels, and $x_{i}$ and $y_{i}$ are corresponding pixels from images x and y. While tested on 330 synthetic images on average the best performance was achieved with the CCC metric. An example comparing the CCC metric with RMSE and SSIM is shown in Figure 3. There are fewer local extrema than with the SSIM metric and the dynamic range is greater (peaks are sharper) compared to the RMS metric. Hence, we applied only the CCC metric for in vivo tests.

### 2.4 Comparison of registration methods using synthetic data

To test the performance of the candidate registration methods, we generated synthetic datasets from 33 previously acquired in vivo 3D FatNav volumes with a 2x2x2 mm^3^ voxel size, obtained during 10 independent scans of 3 volunteers. Each 3D FatNav magnitude image was subjected to uniformly distributed random transformations in ranges ±5, ±10 and ±15 degrees/voxels (both rotations and translations were applied simultaneously) were applied giving 330 pairs of images for each range of motion. Translation was performed using imtranslate (Matlab) for translations, followed by imrotate (Matlab) with cubic interpolation for rotations about the Z, Y and X axes. Any missing pixels in the corners were filled with noise whose mean and SD were matched to values outside the head in the reference image. The reference image is always the first navigator from each dataset – this is usually of best quality because it is always acquired without deliberate motion. This yields a synthetic magnitude dataset. The synthetic datasets were used for testing the speed and accuracy of the registration methods.

### 2.5 In vivo validation

**FIGURE 4:**
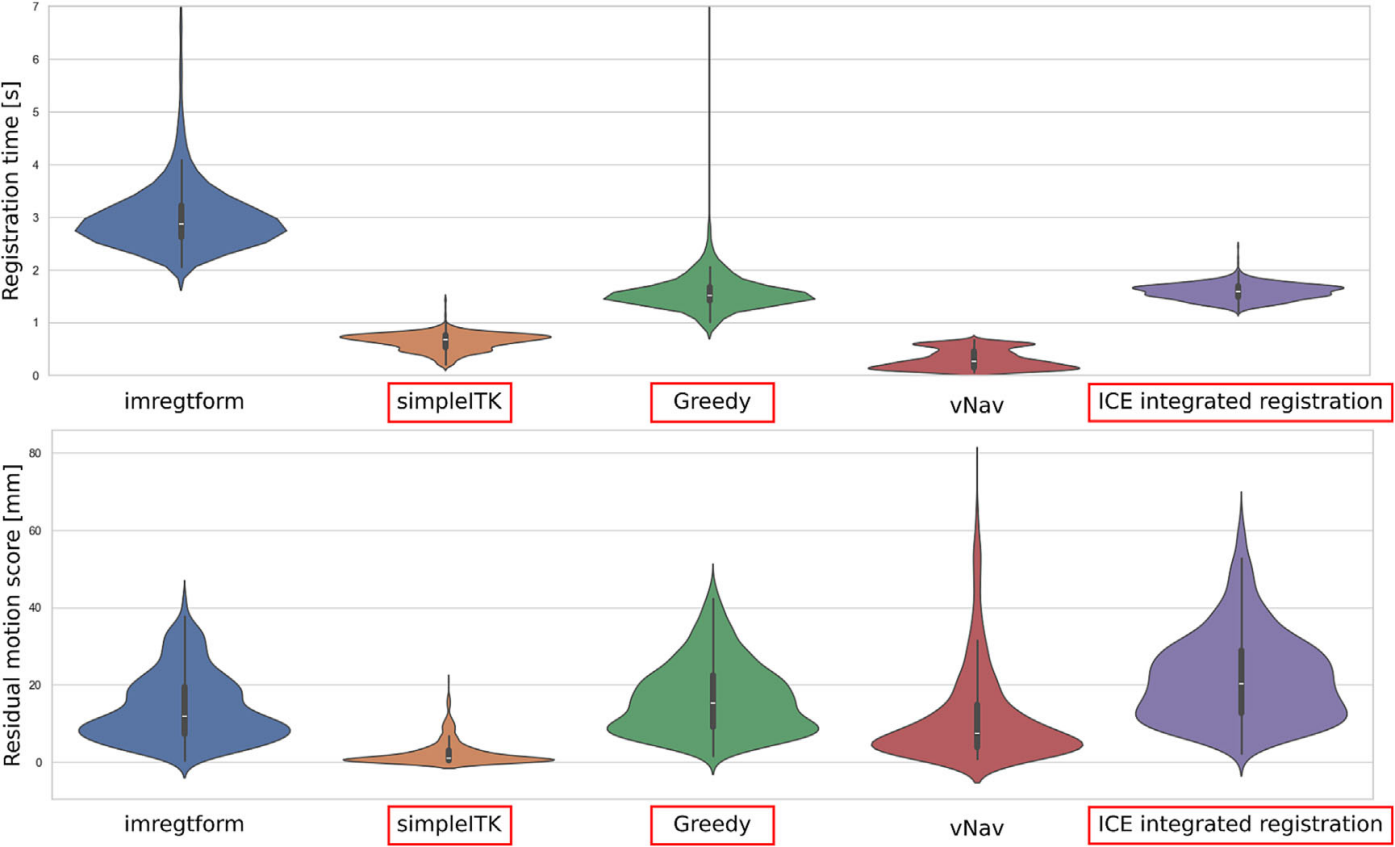
Comparison of speed and accuracy for registration methods on synthetic data comprising 330 3D FatNav image pairs. Full details are given in the methods section. Violin plots of (A) registration time, (B) residual motion score after registration. Smaller motion scores mean that the registration has come closer to the ground truth, i.e. smaller motion scores are better. SimpleITK, greedy and ICE integrated registration (marked with red boxes) were chosen for further assessment in vivo. White dot in the middle of each violin represents the mean value across 330 subjects.

The three best (quickest and most accurate) registration algorithms (SimpleITK, Greedy and ICE integrated algorithm – see Figure 4) were implemented on the scanner with a user‐interface option to select one of them before each scan. SimpleITK and Greedy were installed on an Ubuntu 22.04 LTS Jammy chroot^1^ image that we mounted and ran on the Measurement and Reconstruction System (MARS) computer (the unit responsible for the reconstruction of the images).

Three healthy volunteers gave written informed consent and were scanned with T_1_ and T_2_* weighted 2D GRE protocols. For initial testing, motion updates were set to correct every odd measurement; while every even measurement was acquired in the original reference coordinate frame to allow easy visualisation of the effects of the motion correction. Scan parameters are summarised in Table 1. The volunteer's motion pattern throughout the scan is thus comparable for acquisitions with and without motion correction. All volunteers were scanned with both sequences and with several combinations of the variable parameters shown in Table 2. In all cases, the participant was instructed to change their head position before the acquisition of every other navigator and to try to remain still in between in order to preserve a consistent motion pattern for the corrected and uncorrected host images. The padding used for head immobilisation meant that motion was mainly constrained to nodding in that scan. In scans acquired without pads head movement was possible in all directions. As a final test scans with motion updates for every measurement were also acquired. Here two separate acquisitions were carried out: one with motion correction enabled, and another with motion correction disabled. When motion correction was disabled, we still acquired the FatNav navigator images to determine the motion score. For a fair comparison, we ensured that the images acquired without motion correction had no greater motion score than corrected ones – that is the range of motion during corrected acquisition was at least as big as without correction. Timings of key steps within the motion correction algorithm were recorded for each scan (Figure 2B).

**TABLE 2:** Variable acquisition parameters.

Variable parameters	Options
Deliberate motion	Yes/No
Head immobilisation (chin pads)	Yes/No
Registration method	SimpleITK/Greedy/ICE integrated registration
Motion update frequency	Every scan/every other scan

### 2.6 Quantitative metrics

The range of motion estimated by FatNavs for each measurement was quantified as the Motion Score metric introduced by Tisdall et al [25]. This is defined as

(4) $$\text{Motion score}=∆R+\sqrt{{∆}_{x}^{2}+{∆}_{y}^{2}+{∆}_{z}^{2}},$$

in terms of the rotation‐related displacement ∆R, and the translation‐related displacement ${∆}_{i}$ along the i^th^ axis. This is equivalent to the worst‐case (maximum) displacement of any point on a 64 mm radius sphere, which serves as a simple proxy for an average human head. Higher motion score metric values are worse; lower values are better.

For comparison of the synthetic data with known ground truth values, we also computed the residual motion score, which was calculated as above, but for a transformation, T_residual_ defined as the detected transformation matrix ($T_{d}$) multiplied by the inverted ground truth transformation matrix ($T_{\mathrm{gt}}^{-1}$), i.e. $T_{\text{residual}}=T_{d}\cdot T_{\mathrm{gt}}^{-1}$.

The accuracy of the motion correction was assessed by a standard metric for image sharpness: the variance of Laplacian of the image [26]. This is effective because a Laplace filter detects edges in the image. Hence, its variance tends to increase when the image is sharper because the sharper edges have higher Laplacian values while the flat background continues to have a low Laplacian value. Since the actual Laplacian value depends on what is in the image, we report the ratio of the Laplacian variances of the corrected to uncorrected images.

## 3 RESULTS

### 3.1 Synthetic data

Comparison of the registration algorithms on synthetic data shows that the mean registration time spans from 0.30 s to 2.93 s and the mean residual motion score from 2.83 mm to 19.51 mm. (Figure 4 and Table 3). Matlab's imregtform was excluded as the slowest algorithm. The vNav registration package provides a choice of six registration algorithms and four interpolation options. Initial tests on synthetic data showed that “Algorithm 3” with trilinear interpolation was the fastest and most accurate, hence this configuration was compared with other registration tools. Although this comparison showed that vNav is the fastest, it was excluded at this stage because of its lack of flexibility in accepting input data in the format used within our ICE pipeline. The navigator volume has to be isotropic and each slice is saved in a separate file with a particular naming convention. We could consider the vNav package for future studies once we have adapted its input reader code for our 3D navigator format. The most accurate and reasonably fast registrations were provided by the simpleITK and Greedy algorithms. We included our ICE registration implementation as the third algorithm for in vivo tests because it has the advantage of simplifying deployment of the sequence since no chroot images are required.

**TABLE 3:** Comparison of the registration algorithms on a synthetic dataset comprising 330 FatNav pairs transformed by uniformly distributed random transformations.

Registration method	Average registration time [s]	Average residual motion score [mm]
Imregtform	2.92	14.00
SimpleITK	0.55	2.83
Greedy	1.34	16.81
vNav	0.30	11.61
Ice integrated registration	1.48	19.51

### 3.2 In vivo tests

**FIGURE 5:**
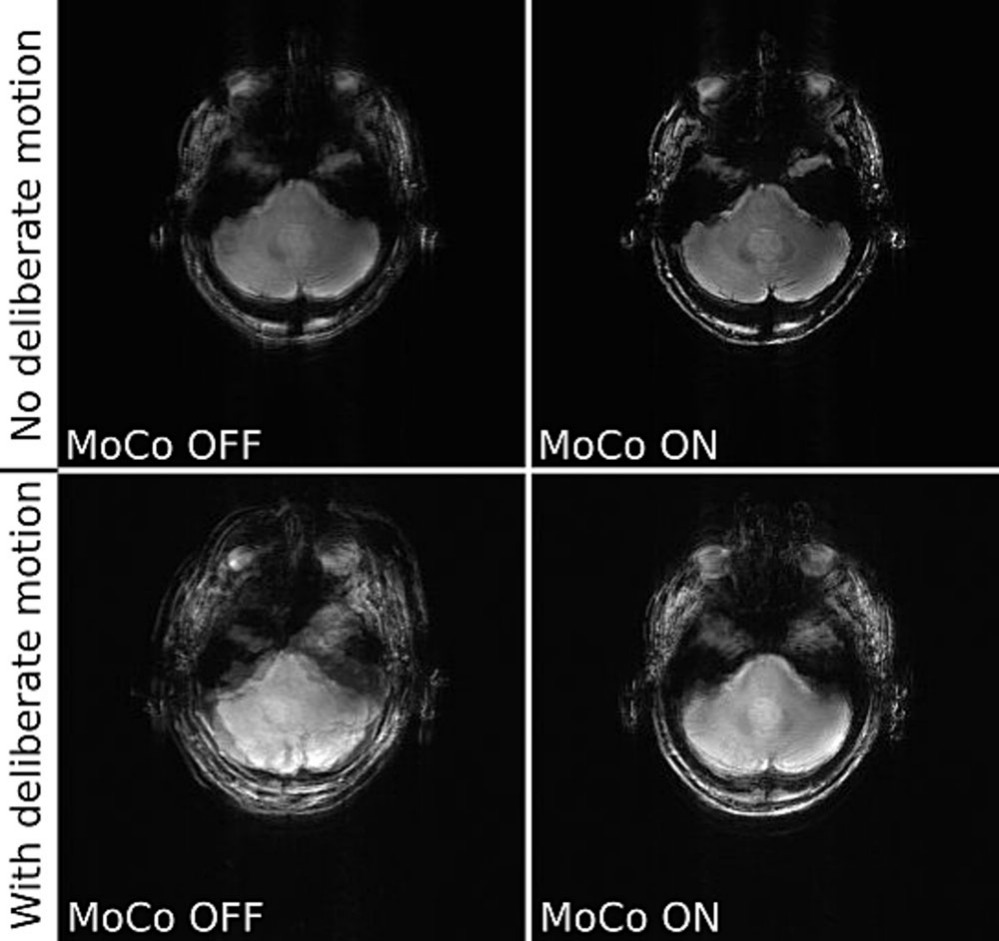
Comparison of uncorrected (MoCo OFF ‐ left) and motion‐corrected (MoCo ON ‐ right) T_1_ weighted GRE images (slice 6/12 in both rows). The subject was a healthy volunteer who was not immobilised. Scans were run without (top row) and with (bottom row) deliberate motion. This data was acquired with ICE integrated registration. The image shown is the sum of the reference scan (first measurement) plus all even numbered measurements (not corrected, left column) or plus all other odd‐numbered measurements (motion corrected, right column). Motion scores describing extent of movement were: 0.56 mm (top row) and 6.95 mm (bottom row), the variance of the image Image sharpness measured by the ratio of the variance of the image Laplacian for corrected vs uncorrected images was: 1.50 (top row) and 0.92 (bottom row).

**FIGURE 6:**
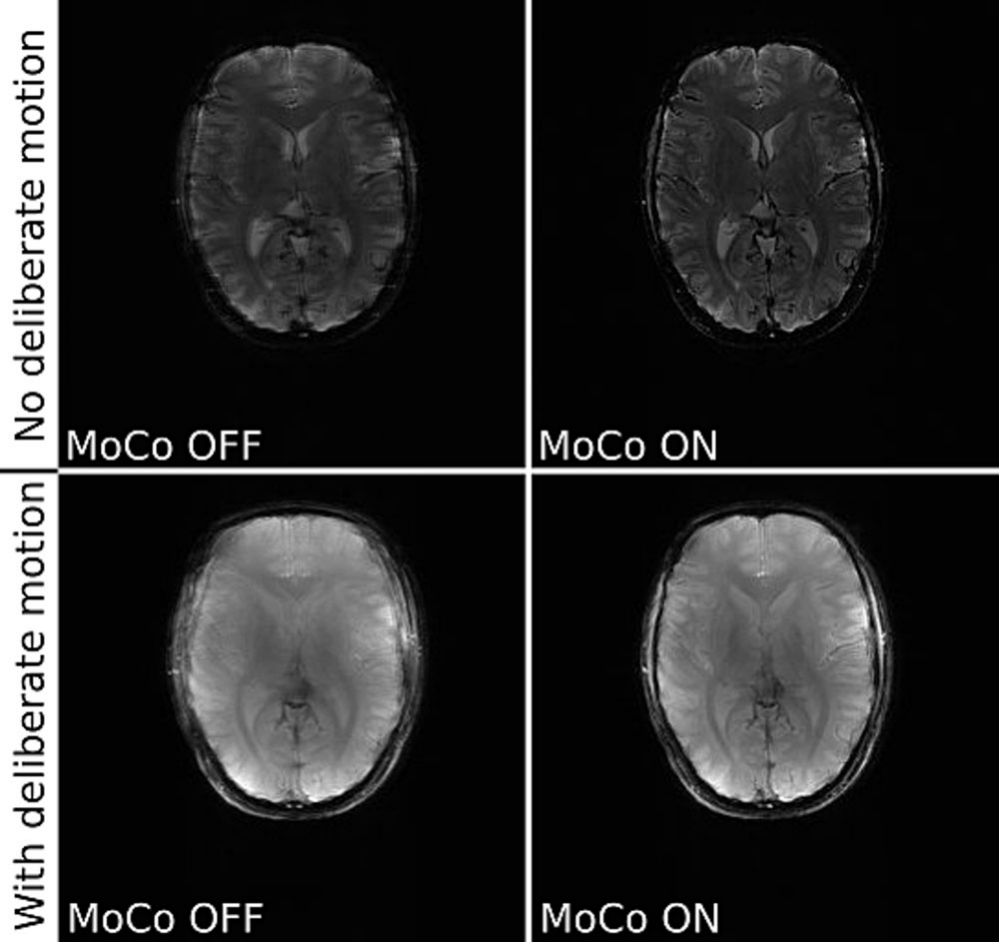
Comparison of not corrected (MoCo OFF ‐ left) and corrected for motion (MoCo ON ‐ right) GRE images of a healthy volunteer (slice 4/10 – top row and 7/12 – bottom row). The top row shows T_2_* weighted images acquired without deliberate motion; the bottom row shows T_1_ weighted images of a volunteer making deliberate movements. Registration was performed with simpleITK. The images shown are sums of either even numbered (not corrected) or odd numbered (corrected) scans added to the first (reference) scan. Motion scores describing extent of movement were: 2.23 mm (top row), 6.55 mm (bottom row), the ratio of the variances of the image Laplacians for corrected vs uncorrected images was: 1.16 (top row), 1.42 (bottom row).

**FIGURE 7:**
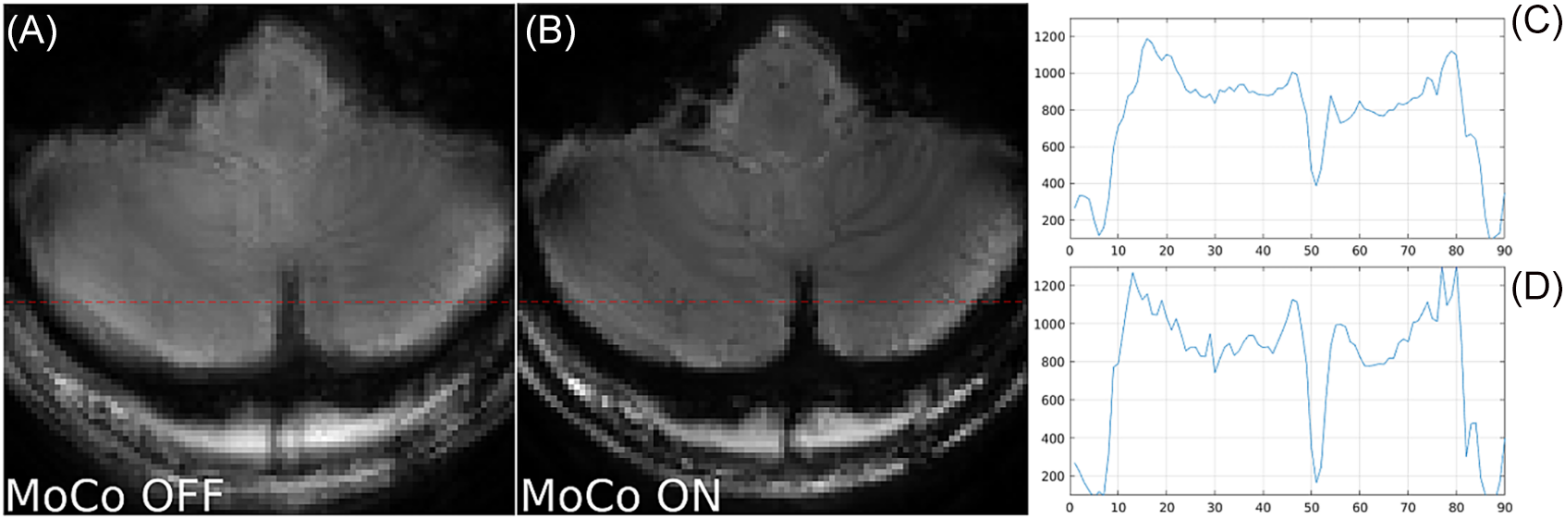
Close up comparison of not corrected (MoCo OFF ‐ A) and corrected for motion (MoCo ON ‐ B) T_1_ weighted GRE images of a healthy volunteer who was asked not to move but who was not immobilised with pads. Note the improved sharpness of vascular structures in the cerebellum and the crisper edges of the brain with motion correction. Red dashed line mark the profile section. Horizontal profiles of not corrected (C) and corrected (B) images. The dip from the middle of the profiles going through cerebral falx was taken to quantify image sharpness (see results). The motion score was: 0.56 mm, the ratio of the variance of the image Laplacian for corrected vs uncorrected was: 1.35.

Figures 5 and 6 compare uncorrected and motion‐corrected images acquired in various conditions using ICE‐integrated and simpleITK registration. We compared the effect of deliberate motion during acquisition with and without head immobilisation. The acquisitions of T_1_ and T_2_* weighted images confirm that our motion correction technique can detect and correct small involuntary motion performed by an experienced volunteer. Even after immobilisation of the head with chin pads some small residual motion occurs and can be corrected, giving crisper image as a result. Deliberate motion without immobilisation is also corrected, but the result is not entirely satisfying for ICE‐integrated registration, which indicates that our algorithm works best in a limited range of motion, below 3.74 mm motion score (Table 4). However, when the subject's head was immobilised – which is clinically more relevant – even substantially exaggerated motion was corrected properly. Note that the edges of the brain and the vascular structure on a close‐up image are better defined in the corrected images (see Figure 7). Profiles across the cerebral falx show that signal intensity drops from 75% to 25% across a distance of 2.75 mm without FatNav but this improves to 1.83 mm with FatNav motion correction. The other two registration methods (simpleITK and Greedy) were able to correct the images with a motion score reaching up to 7.97 mm (Figure 9).

**TABLE 4:** Mean motion scores estimated by ICE online registration.

Sequence	T_1_	T_1_	T_2_*	T_2_*
Deliberate motion	Yes	No	Yes	No
Chin pads	No	No	Yes	Yes
Mean motion score [mm]	6.95	0.56	3.74	0.25

**FIGURE 8:**
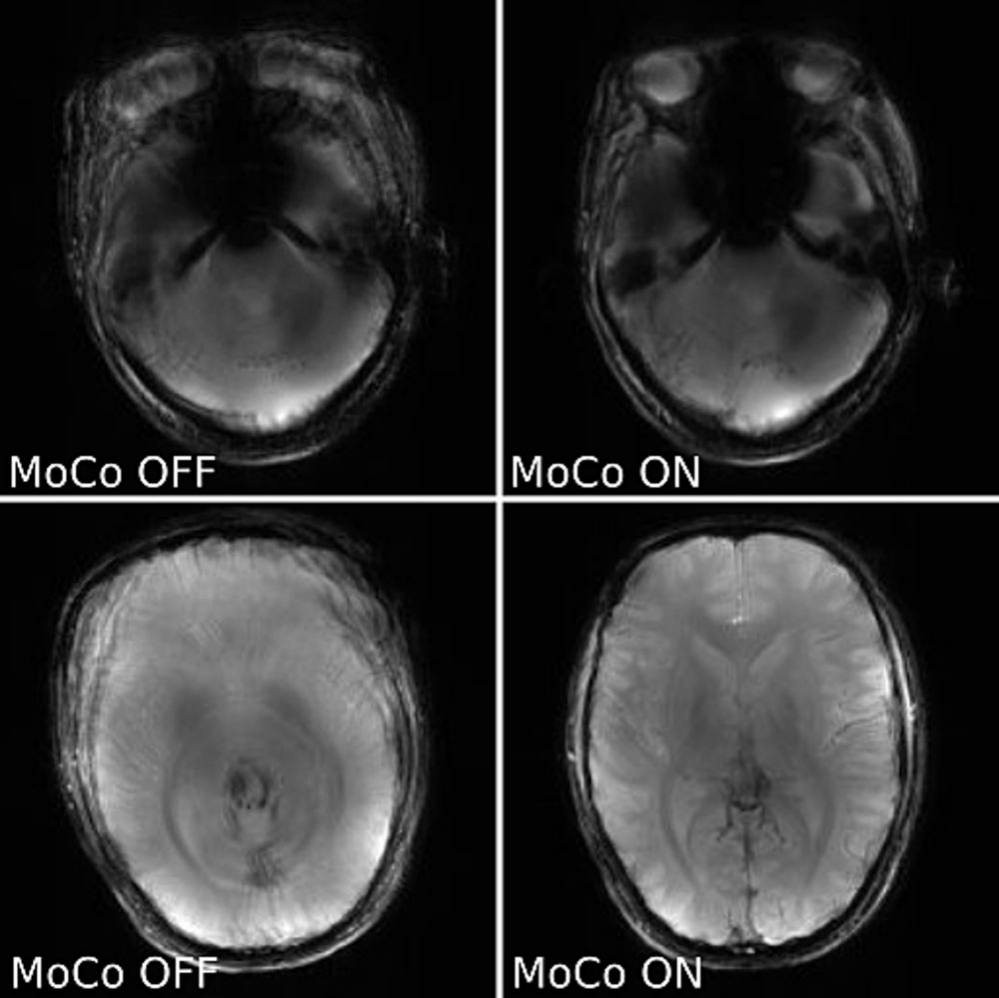
Comparison of T_1_ weighted GRE images acquired for two slices with greedy registration (slice 1/12 – top row, and 6/12 – bottom row). The volunteer was not immobilised and was asked to move deliberately. Images are shown without correction (MoCo OFF ‐ left) and with motion correction (MoCo on ‐ right). Images are sums of all measurements acquired either without or with motion correction. Motion scores describing extent of movement were: 4.53 mm (left) and 6.40 mm (right). The ratio of the variance of the image Laplacian for corrected vs uncorrected was: 0.91 (top) and 1.59 (bottom).

**FIGURE 9:**
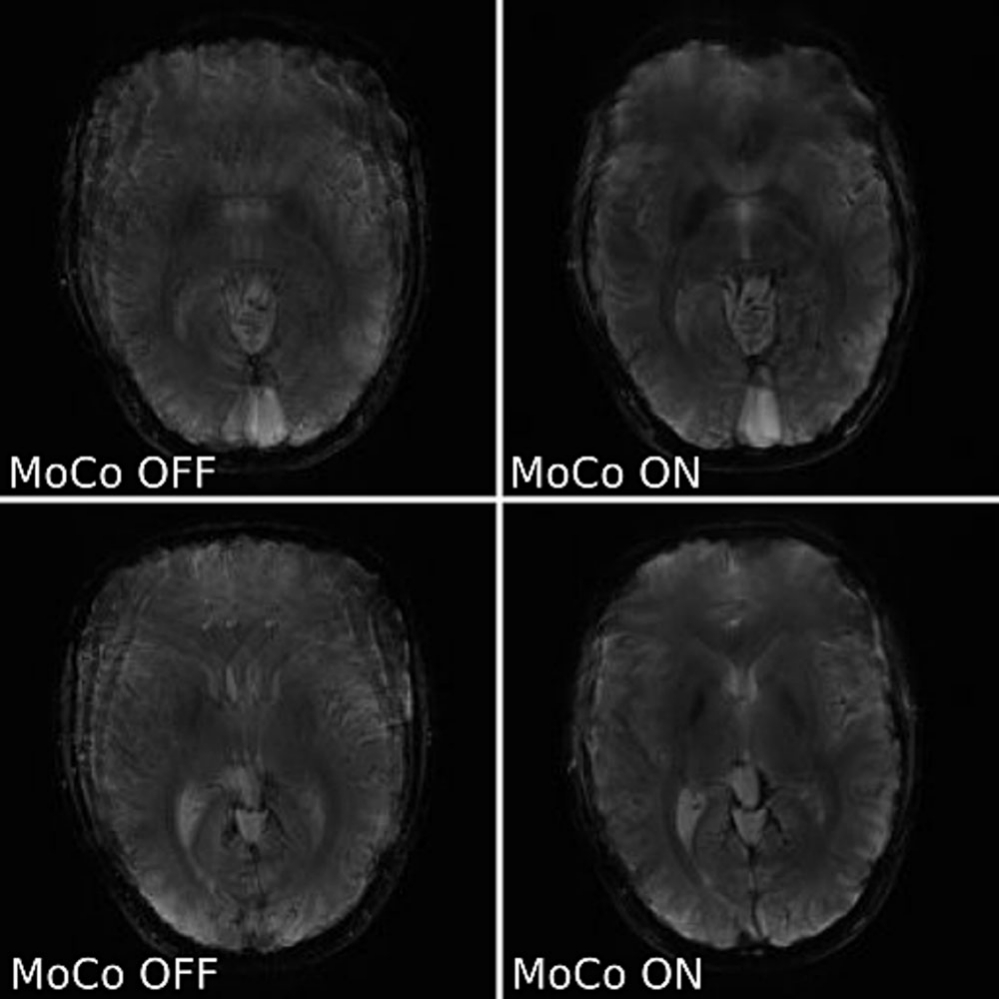
Comparison of two slices of not corrected (left) and corrected for motion (right) T_2_* weighted GRE images of a not immobilised healthy volunteer with deliberate motion (slice 4/10 – top row, and 6/10 – bottom row). These images used greedy registration. The images shown are sums of all measurements acquired either without (MoCo OFF ‐ left) or with motion correction (MoCo ON ‐ right). Motion scores describing extent of movement were: 5.07 mm (left, not corrected) and 7.97 mm (right, motion corrected). The ratio of the variance of the image Laplacian for corrected vs uncorrected was: 1.01 (top) and 1.07 (bottom).

In a final test, the motion updates were done for every measurement (Figures 8 and 9). The motion scores describing extent of movement were greater for acquisitions corrected for motion, which means these cases were more difficult to correct. The volunteers were asked to change pose before each navigator, but it did not prevent occurrence of residual motion, which was likely, because head wasn't immobilised.

The timing diagram (Figure 2B) splits the whole procedure of motion correction into four events. Once the motion parameters are estimated, the actual sequence update takes a maximum of 2 ms. This small delay occurs when the motion update parameters arrive during acquisition of a particular *k*‐space line, so the effective motion update is delayed until the next *k*‐space line. The most time‐consuming steps are acquisition, reconstruction and registration of the navigators. Note that registration times logged on the scanner differ from those for synthetic data because of the different performance of the MARS and the desktop computer used for synthetic data tests. Also on the scanner, simpleITK and Greedy are both slowed down by data conversion to their required input formats, while the ICE‐integrated registration benefits from working directly on the data in memory. The average registration times recorded during in vivo scans were: 1.29 s, 1.32 s and 1.05 s for simpleITK, Greedy and ICE integrated registration, respectively.

The acquired navigators contain information about the actual position of the head during imaging which not only allows for calculation of the motion score but also can be used to improve correction retrospectively in case the prospective update did not eliminate all motion. Such approach, although with some limitations, can work for the presented here case of 2D host sequence with a 3D navigator [27].

## 4 DISCUSSION

The images we obtained confirm the value of prospective 3D FatNav for correcting clinically relevant ranges of head motion in 7T MRI. The test paradigm with deliberate motion before every other imaging block of interleaved sequence updating every other measurement for motion gives realistic results and eliminates concerns about the reproducibility of the motion pattern in two separate measurements with and without motion correction. We used a relatively slow update rate for the acquisition of the presented results to help ensure the volunteers were still for the reference and test images in each pair.

If we need to correct more frequent motion, the current implementation can make updates approximately every 2 x TR. The most time‐consuming element is FatNav acquisition which takes T_Navacq_ = 1.23 s. The ensuing online reconstruction and registration requires approximately 2.5 s. Hence, for sequences with TR ~ 2.5 s, it is possible to update motion parameters within two *k*‐space lines. However, that would elongate the total acquisition time by N * T_Navacq_. Hence, we chose to make a motion update once before each measurement. This seems to be sufficient for compliant patients, but the update rate could be increased at a cost of total acquisition time if needed.

In all cases tested, prospective FatNav motion correction improved image quality. This applied even for motion substantially larger than expected in clinical practice. Retrospective manual registration revealed that the most challenging situations were when significant rotation occurred about an oblique axis. This stems from the implementation of the ICE‐integrated registration algorithm which searches around each principal axis in turn. This was a trade‐off between speed and accuracy. Greedy and simpleITK registration were more robust for larger‐scale motions. However, we note that methods for rapidly correcting modest motion are still valuable [28].

We believe that ICE‐integrated registration is quick and accurate enough for compliant patients. For patient populations who are less compliant (e.g. patients with dementia) it may be preferable to use Greedy or simpleITK registration online.

The corrected T_2_* weighted images can potentially reveal small changes, like microbleeds which could be smoothed out even by small involuntary motion of immobilised patients.

The presented implementation is flexible and modular enough to be implemented in most of the clinically used sequences. We also recently showed that the same framework can be used to make online B_0_ correction for MR spectroscopy [18, 29, 30].

A similar implementation of FatNav prospective motion correction for Philips scanner confirms the usefulness of this method for compliant volunteers [13]. They acquired improved images of cerebellar cortical layers in 0.19 mm resolution. We confirmed this result in lower resolution showing that prospective FatNavs can improve image sharpness in the cerebellum by correcting residual motion of the volunteer trying to remain still while scanning.

One limitation of this study is the metric for image sharpness. We chose the variance (between pixels) of the image Laplacian. However, we observed that for cases showing severe motion artefacts, the artefacts could sometimes contribute so strongly to the variance of the Laplacian that this obscured the loss of sharpness for the head itself. Hence sometimes FatNav corrected images had lower variance of Laplacian even though it was clear on visual inspection that they had improved image sharpness and reduced motion artefacts.

Another limitation is that the 3D FatNav acquisition is relatively slow at 1.23 s. Furthermore, it is a relatively high spatial resolution which increases the time for image reconstruction and coregistration too. We achieved a navigator acquisition to sequence update time of approximately 4 s which has been reported as being acceptable for retrospective motion correction [31].

We carried out preliminary tests with lower‐resolution FatNavs to assess whether they provide a better trade‐off between motion update accuracy and timeliness. We decreased the navigator resolution to 32x32x32, 16x16x16 and 8x8x8 and compared acquisition, reconstruction and registration times and the accuracy (expressed as residual motion score). The residual motion score increases by less than 10 mm for 32x32x32 and 16x16x16 navigators as shown in Figure SI 1a in the Supplementary Information. With a lower resolution, registration times are below 400 ms for the four registration methods that we tested as shown in Figure SI 1b in the Supplementary Information. The total motion update time is less than 1 s for 16x16x16 and 8x8x8 navigators as shown in Figure SI 1c in the Supplementary Information. We propose that a 16x16x16 resolution FatNav strikes a good balance between speed and accuracy.

Inserting more than one FatNav per host image might affect the quality of the host images by disturbing the magnetisation steady state. We inserted three different resolutions (128x128x88, 32x32x32 and 16x16x16) FatNavs (one at a time) into a single slice host image of 128x128 resolution every *N* ^th^ line of k‐space. The resulting host images are shown in Figure SI 2 in the Supplementary Information. Some ghosting is visible with FatNavs every 4th or every 2nd line of k‐space for a single slice host GRE scan. We repeated this test for a multi‐slice GRE scan as host image. Figure SI3 in the Supplementary Information shows that there is a negligible level of ghosting with 16x16x16 FatNavs inserted every 16th or every 128th line of k‐space.

In future work beyond the scope of this study, we hope that our framework can be used with small modifications to implement collapsed 2D navigators registered to the 3D reference volume. This approach has been reported to decrease navigator acquisition time below 30 ms at 3T [32, 33].

Further improvement of the accuracy of the presented technique may require addition of the B_0_ correction; especially for the T_2_* sequence [34]. The benefit of FatNav over the vNav implementation presented by Liu et al is that fat‐selective excitation does not disturb water signal. On the other hand, due to the presence of the fat signal mostly at the scalp, it would not be practical to use dual echo B_0_ mapping based on FatNavs, and the B_0_ correction would require additional readouts.

## 5 CONCLUSION

We have successfully implemented prospective 3D FatNav motion correction for 7T Terra MRI scanners. The corrections improve image quality in scans with deliberate motion. To the best of our knowledge, this is the first implementation of self‐contained (not using external hardware) prospective motion correction for the Siemens 7T Terra. Our FatNav Sequence Building Block and ICE implementation are available to collaborators subject to the usual formalities.

## CONFLICT OF INTEREST STATEMENT

Iulius Dragonu is an employee of Siemens Healthcare LTD, Camberley, UK.

Christopher T. Rodgers receives a research grant from Siemens for a different project.

## ETHICS STATEMENT

All volunteers scanned in the study gave written informed consent.

## Supporting information


**Figure S1.** Comparison of accuracy and timing of FatNavs acquired at the original Gallichan et al resolution (128x128x88) and three lower resolutions (32x32x32, 16x16x16, 8x8x8). a – residual motion scores navigators registered with the methods described in the manuscript, Greedy registration of the high‐resolution FatNav was taken as the ground truth, thus the motion score is zero in this case. b ‐registration times of the navigators. c – total motion update time (acquisition + reconstruction + registration) for navigators acquired at different resolutions.

**Figure S2.** Single slice host image (128x128 pixels) acquired with FatNavs inserted every *N* ^th^ k‐space line of the host acquisition. Navigators were tested at three resolutions: hr128: high resolution 128x128x88, iso32: isotropic 32x32x32 and iso16: isotropic 16x16x16. With typical windowing of the host image showing broad range of intensities, a ghosting artefact can be noticed for N = 2 and N = 4 acquisitions.

**Figure S3.** Multislice host images (128x128 pixels in‐plane resolution) acquired with FatNavs (iso16: 16x16x16) inserted every Nth k‐space line of host acquisition (top row: N = 16, bottom row N = 128).

## Data Availability

The data that support the findings of this study are available on request from the corresponding author. The data are not publicly available due to privacy or ethical restrictions.

## ENDNOTE

1 Chroot command allows to switch to another root file system on linux platforms. This means that inside chroot directory additional applications with all necessary dependencies can be installed without interfering with the main OS.
