# Supplementary figures and images for: Prospective 3D Fat Navigator (FatNav) motion correction for 7T Terra MRI
Source: NMR Biomed. 2024 Oct 26;38(1):e5283. doi: 10.1002/nbm.5283 (PMC11602639; doi:10.1002/nbm.5283)

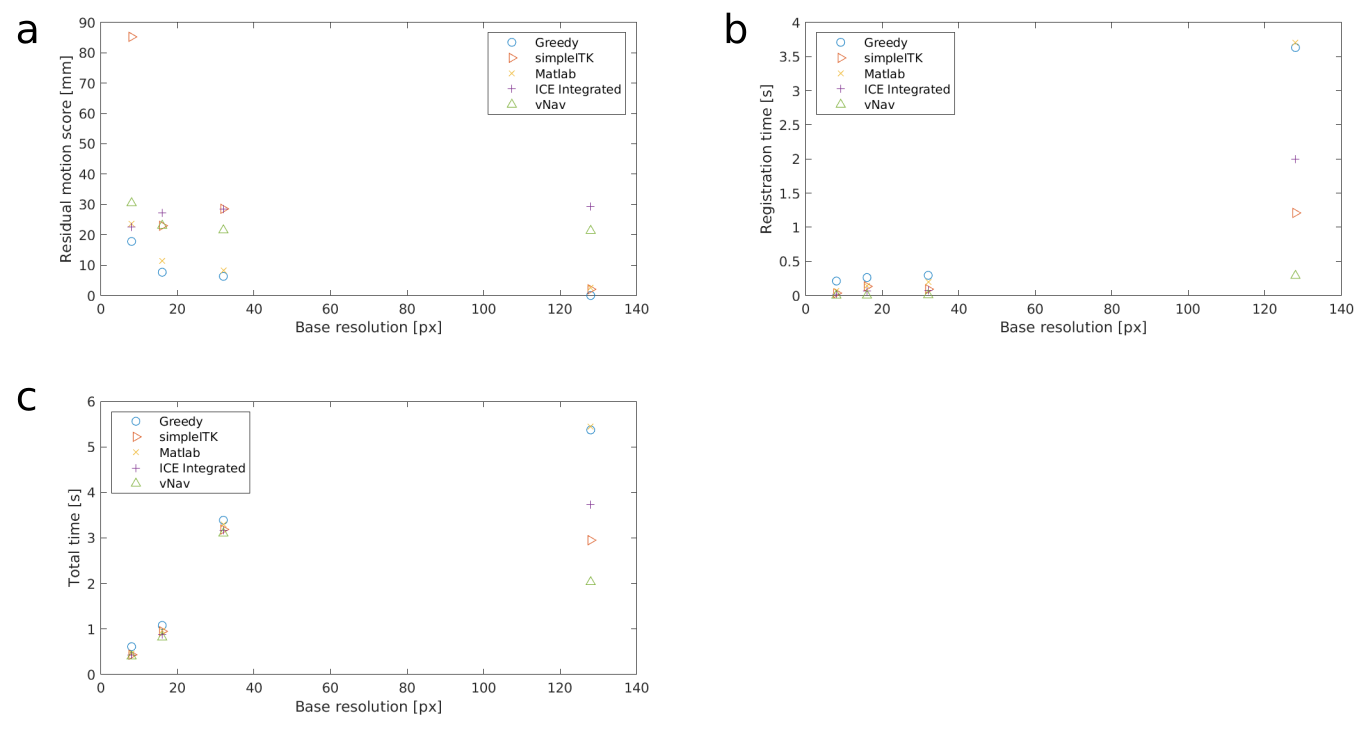

Supplement: Supplementary file 1 — Figure S1. Comparison of accuracy and timing of FatNavs acquired at the original Gallichan et al resolution (128x128x88) and three lower resolutions (32x32x32, 16x16x16, 8x8x8). a – residual motion scores navigators registered with the methods described in the manuscript, Greedy registration of the high‐resolution FatNav was taken as the ground truth, thus the motion score is zero in this case. b ‐registration times of the navigators. c – total motion update time (acquisition + reconstruction + registration) for navigators acquired at different resolutions. [file NBM-38-e5283-s002.png]

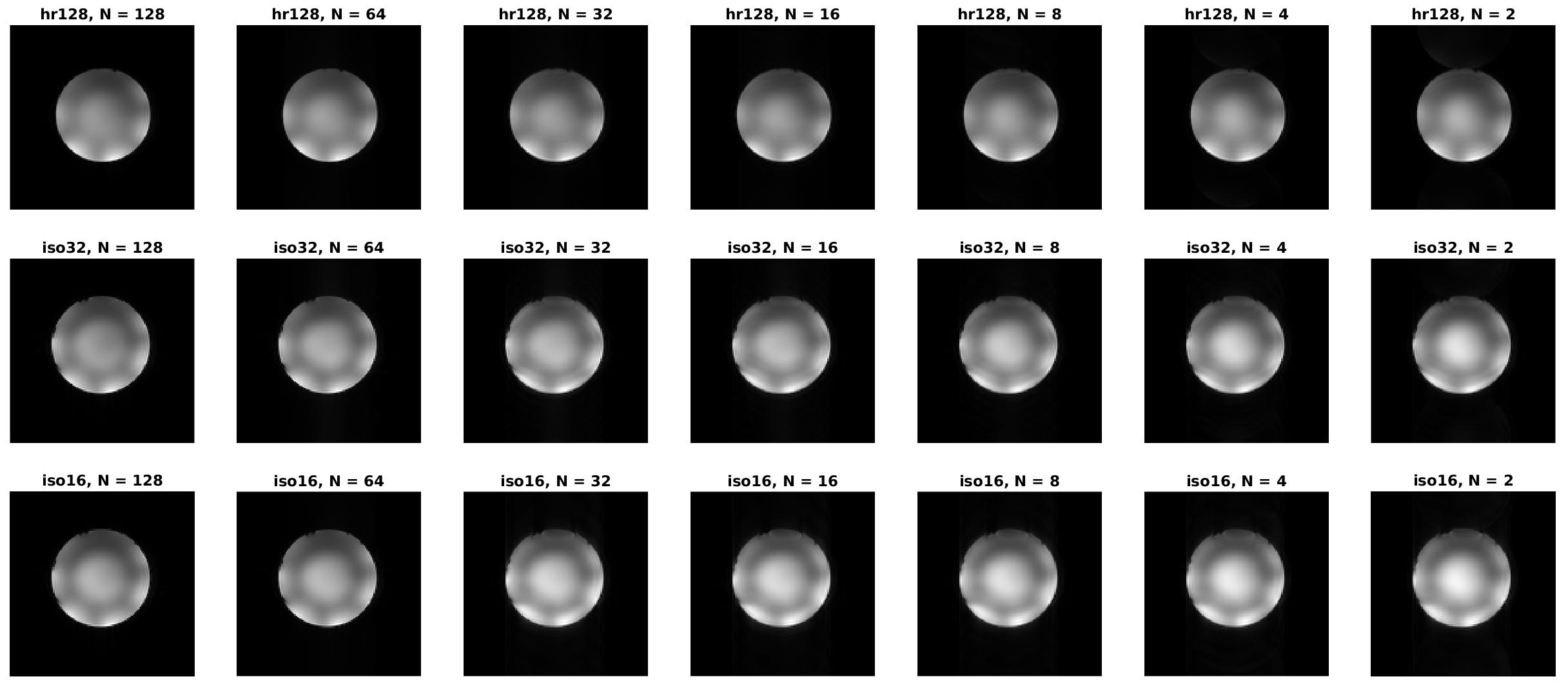

Supplement: Supplementary file 2 — Figure S2. Single slice host image (128x128 pixels) acquired with FatNavs inserted every N th k‐space line of the host acquisition. Navigators were tested at three resolutions: hr128: high resolution 128x128x88, iso32: isotropic 32x32x32 and iso16: isotropic 16x16x16. With typical windowing of the host image showing broad range of intensities, a ghosting artefact can be noticed for N = 2 and N = 4 acquisitions. [file NBM-38-e5283-s001.png]

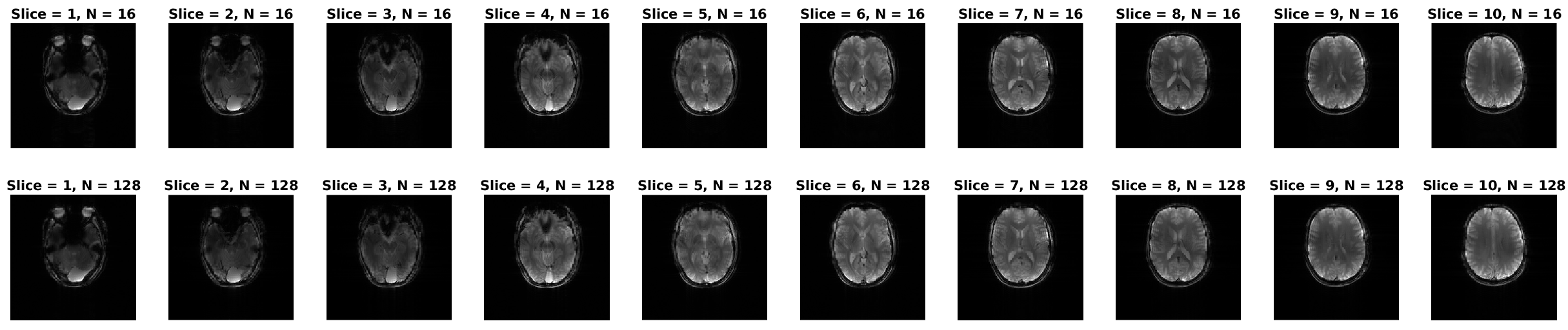

Supplement: Supplementary file 3 — Figure S3. Multislice host images (128x128 pixels in‐plane resolution) acquired with FatNavs (iso16: 16x16x16) inserted every Nth k‐space line of host acquisition (top row: N = 16, bottom row N = 128). [file NBM-38-e5283-s003.png]
